# Spatial-temporal dynamics of carbon emissions and carbon sinks in economically developed areas of China: a case study of Guangdong Province

**DOI:** 10.1038/s41598-018-31733-7

**Published:** 2018-09-06

**Authors:** Jie Pei, Zheng Niu, Li Wang, Xiao-Peng Song, Ni Huang, Jing Geng, Yan-Bin Wu, Hong-Hui Jiang

**Affiliations:** 1 The State Key Laboratory of Remote Sensing Science, Institute of Remote Sensing and Digital Earth, Chinese Academy of Sciences, Beijing, 100101 P.R. China; 2 University of Chinese Academy of Sciences, Beijing, 100049 P.R. China; 3 College of Management Science and Engineering, Hebei University of Economics and Business, Shijiazhuang, 050061 P.R. China; 4 Department of Geographical Sciences, University of Maryland, College Park, Maryland 20742 USA; 5 Key Laboratory of Ecosystem Network Observation and Modeling, Institute of Geographic Sciences and Natural Resources Research, Chinese Academy of Sciences, Beijing, 100101 China; 6 Key Area Planning Construction and Management Bureau of Longgang, Shenzhen, Shenzhen, 518116 P.R. China

## Abstract

This study analysed spatial-temporal dynamics of carbon emissions and carbon sinks in Guangdong Province, South China. The methodology was based on land use/land cover data interpreted from continuous high-resolution satellite images and energy consumption statistics, using carbon emission/sink factor method. The results indicated that: (1) From 2005 to 2013, different land use/land cover types in Guangdong experienced varying degrees of change in area, primarily the expansion of built-up land and shrinkage of forest land and grassland; (2) Total carbon emissions increased sharply, from 76.11 to 140.19 TgC yr^−1^ at the provincial level, with an average annual growth rate of 10.52%, while vegetation carbon sinks declined slightly, from 54.52 to 53.20 TgC yr^−1^. Both factors showed significant regional differences, with Pearl River Delta and North Guangdong contributing over 50% to provincial carbon emissions and carbon sinks, respectively; (3) Correlation analysis showed social-economic factors (GDP per capita and permanent resident population) have significant positive impacts on carbon emissions at the provincial and city levels; (4) The relationship between economic growth and carbon emission intensity suggests that carbon emission efficiency in Guangdong improves with economic growth. This study provides new insight for Guangdong to achieve carbon reduction goals and realize low-carbon development.

## Introduction

Global warming, one of the most serious environmental issues humans must currently confront, is due primarily to greenhouse gas (GHG) emissions^1^. Land use/land cover change (LULCC) is the second largest source of GHG emissions, after fossil fuel combustion^2^. LULCC accounts for 25% of all carbon emissions since industrialization^3^. While the contribution of LULCC to anthropogenic carbon emissions has been decreasing since 2000^4^, the decreasing proportion is mainly attributed to the increase in emissions from fossil fuel combustion^5^. Earth system models and observation-based methods are commonly used in estimating carbon emissions from LULCC^6^. However, uncertainties in data and differences in considering LULCC activities create considerable uncertainty^5^. Since it is difficult to assess the accuracy of historical LULCC data before the satellite era, which were constructed based on nationally and internationally aggregated land-use statistics^5^, and the interpretation and classification of remotely sensed images might be influenced by human subjectivity, variations in LULCC emissions estimates will emerge when using different LULCC data sets. For example, Shevliakova *et al*.^7^ estimated that a difference of 0.2 Pg-C yr^−1^ was produced when using two different spatial data sets for croplands (the SAGE and HYDE data sets). Furthermore, variations also originate from inclusion of different LULCC processes (e.g. forest management)^8^.

Satellite-based observations of LULCC provide an alternative to estimate spatiotemporal variations of carbon emissions^9,10^. To date, the spatial resolution of existing global land cover data sets has improved from 1 km to 30 m^11–15^. However, these global land cover products lack interoperability because they were produced using different classification systems and algorithms^16,17^. Furthermore, they are not continuous in time and their accuracies may not always satisfy the requirements of research on a local scale. It is possible to solve these problems and produce more accurate results of spatiotemporal patterns of LULCC in study areas by using digitally enhanced, multi-sensor satellite images with high spatial resolution produced with a standard classification system over several consecutive years.

Extensive research has been done on LULCC-related carbon emissions at the global, regional, and national spatial scales^10,18–22^. Those studies presented various views on the relationships between carbon emissions and land use by discussing natural carbon processes of LULCC and the impacts on anthropogenic carbon emissions by LULCC. The carbon dynamics caused by LULCC could be investigated through the division of different land use/land cover types, and carbon emission effects of different land use/land cover types could thus be accurately explored^23^. However, previous studies were mainly focused on carbon emissions taking place on a single land-use type, such as carbon emissions resulting from agricultural land utilization^24^ or built-up land construction^25^, neglecting to establish the whole carbon estimation system based on land use/land cover^23^. Furthermore, a comprehensive comparison between carbon emissions and carbon sinks of land use/land cover remains lacking. Meanwhile, due to data limitation, there were few reports on continuous time series estimation of regional land-use carbon emissions, which is of critical importance for local governments to formulate or adjust carbon reduction policies in a timely manner^23^.

It has been reported that coastal regions are an ideal study area for exploring the relationship between land use patterns and carbon emissions derived from social and economic factors, since these regions are characterized by high population densities, rapid economic growth and intense energy consumption^26–28^. Guangdong, the most affluent and populous province in China, has undergone rapid development since the implementation of China’s economic reform and opening policy in 1978^29,30^. The gross domestic product (GDP) of Guangdong has steadily increased, with an average annual growth of 18% for the last three decades, reaching 1.197 trillion USD in 2016. Nevertheless, in economically developed regions like Guangdong Province, due to the relatively high degree of urbanization, although LULCC during the most recent years is not dramatic compared with other rapidly developing regions, carbon emissions of land use are still very large. This is because a considerable amount of carbon emissions could be originated from many sources in addition to LULCC, including mechanized agricultural tillage and urban construction driven by human economic activities^23,31^. Both are associated with carbon emissions resulting from energy consumption and industrial production. As reported, Guangdong’s high-speed economic development relies heavily upon energy consumption, 21.9 million tons of standard coal was consumed in the province in 2010, with the amplitude of the annual growth in energy consumption close to 11% since the year 2000^32^. Large increases in energy consumption lead to large increases in carbon emissions. However, the carbon emissions of energy and industrial sources also indirectly come from land use carbon emissions when taking into account the demand that population growth and economic development have induced for more lands converted to construction use^33^. Therefore, the regional land use carbon emissions should be analysed to determine how to reduce carbon emissions through land use policy^34,35^.

Previous studies of carbon emissions in Guangdong Province were either focused on energy-related carbon emissions or terrestrial ecosystem carbon stock dynamics^29,36,37^. However, few studies comprehensively estimated multi-scale land use carbon emissions and its relationship with social-economic factors in this region. To bridge this gap, in this study, we attempted to realize a complete accounting of carbon dynamics in Guangdong Province on multiple spatial scales, from 2005 to 2013, by using continuous land use/land cover data with high spatial resolution (≤30 m) combined with energy consumption statistics. This may also help to understand the relationship between urbanization and land use carbon emissions^38^. The specific objectives of this paper are: (1) to examine the area of different land use/land cover types in Guangdong by visually interpreting multi-sensor high-resolution satellite images for nine consecutive years based on a standard classification system and interpretation symbol library; (2) to investigate spatiotemporal changes in vegetation carbon sinks and carbon emissions on a provincial, sub-provincial and city scale; (3) to explore the relationship between carbon emissions and social-economic factors on a provincial and city scale; (4) to quantitatively study spatiotemporal changes in carbon emissions intensity (CEI) and its relationship with economic growth.

## Methods

### Study area

**Figure 1:**
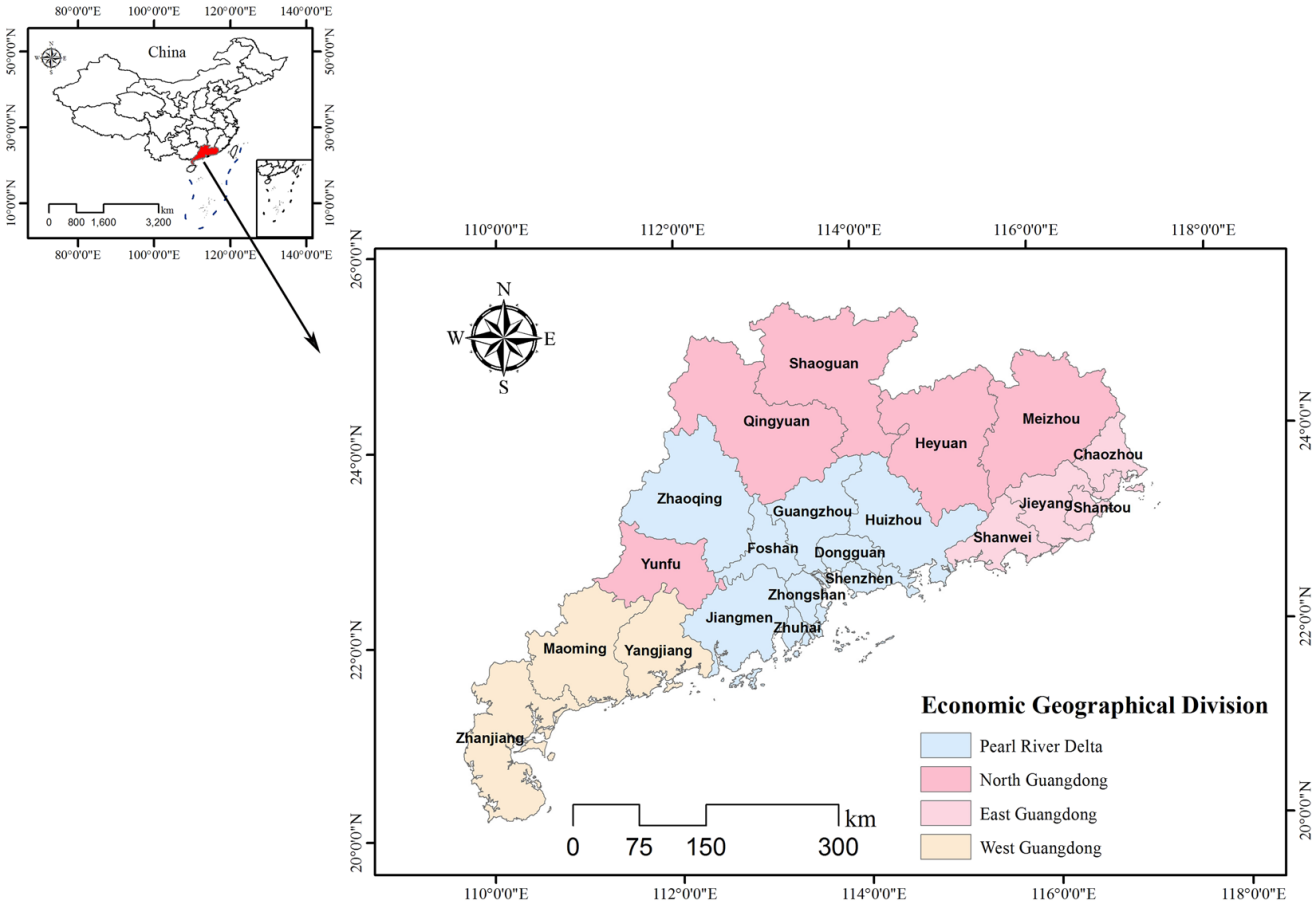
Location of Guangdong Province in China, and the economic geographical division of Guangdong. Map created using ArcGIS [9.3], (http://www.esri.com/software/arcgis).

Guangdong Province (20°13′–25°31′N, 109°39′–117°19′E; Fig. 1) is in the southernmost part of the Chinese mainland. The terrain is generally high in the north and low in the south, incorporating plains, hills, mountains, and plateaus. East Asian monsoon is the main climatic type in this area. Guangdong is one of the richest regions in light, heat, and water resources in China. The average annual temperature is 21.8 °C and the average annual rainfall is 1789.3 mm. The permanent resident population is 108 million, of which 68.71% is urban (2015). The real GDP has been the highest in China for the last 27 years, and was 1.197 trillion USD in 2016^39^. Guangdong is divided into four regions by geography and economic level^40^: Pearl River Delta (PRD), East Guangdong (EGD), West Guangdong (WGD), and North Guangdong (NGD). The cities included in each region are shown in Fig. 1.

### Data sources

Three forms of data were used in this study, specifically, spatial data, statistical data and empirical coefficients. Spatial data included highly qualified annual land use/land cover data (from the consecutive years 2005–2013) that were derived from multi-source satellite images, composed of CBERS and Landsat series satellite images at a fine spatial scale (≤30 m). The specific descriptions of yearly satellite images acquired from 2005–2013 are shown in Supplementary Table S1. The statistical data included energy consumption data, agricultural practice-related statistics and social-economic data. Specifically, annual energy consumption data were obtained from “Guangdong Statistical Yearbook” and “China Energy Statistical Yearbook”. Agriculture-related statistical data included consumption of chemical fertilizers, pesticide, plastic film for farm use, total power of agricultural machinery, effective irrigation area, and sown area of major farm crops, and these data were acquired from “Guangdong Statistical Yearbook” and “China Agriculture Statistical Yearbook”, complemented by the annual statistical yearbooks of 21 cities in Guangdong. Social and economic data, including population and GDP, originated from “Guangdong Statistical Yearbook”. Empirical coefficients, including carbon emission factors and carbon sink factors, were taken from relevant literature.

### Derivation of land use/land cover data

Remote sensing technology was used to interpret the area of main land use/land cover types from 2005 to 2013. Firstly, a collection of multi-temporal satellite images was selected. Data pre-processing was then conducted, including radiometric calibration, atmospheric correction, geometric correction, band composite, and image mosaic. To detect changes in land use/land cover, multi-temporal images were co-registered in the same coordinate system. A standard classification system (i.e. National Ecological Remote Sensing Monitoring Land Use/Land Cover Classification System) was determined to ensure multi-source images were classified consistently. The land use/land cover classifications included six first-level classifications and 25 second-level classifications (Supplementary Table S2). To improve the interpretation accuracy, a multi-temporal interpretation symbol library of land use/land cover types was established. Visual interpretation was applied on multi-source remotely sensed images to achieve land use/land cover data of Guangdong Province from 2005 to 2013. Finally, field investigations were made to validate the accuracy. For the purposes of this study, we mainly focused on first-level classification types. To identify the transfer between different land types, a land use transition matrix between 2005 and 2013 was created using intersect analysis tool in ArcGIS 9.3^31^.

### Carbon sink estimations from forest land and grassland

Forest land and grassland contribute most of the gain to vegetation carbon storage in Guangdong, due to photosynthesis. While farm crops absorb CO_2_ from the atmosphere while growing, most of the increased biomass is released into the atmosphere through decomposition in the short term^41–43^. Therefore, cropland was not included in the calculation. Due to data limitation, carbon sinks of water bodies and barren land were also excluded. With reference to existing research^23,31^, vegetation carbon sinks of land cover were measured by using carbon sink factors and corresponding land cover area, which provided a simple and practical method for the calculation of multi-scale carbon sinks. The formula for estimating the annual carbon sinks of land cover from 2005 to 2013 is shown in Eq. (1):

1 $${C}_{veg}=\sum _{i}{R}_{veg \mbox{-} i}\times Are{a}_{veg \mbox{-} i}$$

where *C*_*veg*_ represents annual total carbon sinks; *R*_*veg*-*i*_ refers to carbon sink factors of different land cover types (i.e. forest land and grassland, respectively), which were quoted from relevant research conducted in Guangdong (Supplementary Table S4). To improve the accuracy of estimation, different values of carbon sink factor of the same land cover type were averaged. Meanwhile, considering the environmental conditions are relatively consistent in the whole study area, carbon sink factor of the same land cover type in different regions/cities remains unchanged. *Area*_*veg*-*i*_ is the area of forest land and grassland, respectively.

### Carbon emissions estimation from cropland utilisation

Carbon emissions from cropland utilisation refer to the direct and indirect carbon emissions resulting from agricultural production activities based on cropland resources^24^. Rice paddies are one of the primary emitters of methane (CH_4_) to the atmosphere^44,45^, which is regarded as the second most important trace GHG after CO_2_^46^. Two rice crops are grown in Guangdong Province each year under irrigation, causing the CH_4_ emissions in this area to be relatively higher than other rice cultivation regions of China^46^. Meanwhile, in Guangdong, water is usually drained out from paddy fields during the non-cropping season, and the amount of CO_2_ released into the atmosphere will significantly increase in this period^47^. Therefore, CH_4_ emissions in the flooding period and CO_2_ emissions in the non-flooding period were viewed in this study as two direct carbon emission processes for cropland. Referring to the calculation method adopted by IPCC^48^, CH_4_ emissions from paddy fields were estimated by paddy area and CH_4_ emission factor. Similarly, CO_2_ emissions during non-growing season were calculated by paddy area and CO_2_ emission factor. The specific formula for estimating direct carbon emissions from agricultural activities is as follows (Eq. (2)):

2 $$C{E}_{crop \mbox{-} dir}=Are{a}_{paddy}\times ({C}_{C{H}_{4} \mbox{-} C}\times \frac{12}{16}+{C}_{C{O}_{2} \mbox{-} C}\times \frac{12}{44})$$

where *CE*_*crop-dir*_ denotes annual direct carbon emissions from agricultural activities; *Area*_*paddy*_ refers to area of paddy fields, which was derived from interpretation of satellite images; *C*_*CH4-C*_ and *C*_*CO*2*-C*_ represent CH_4_ emission factor and CO_2_ emission factor, respectively. The value of *C*_*CH4-C*_ (0.76 Mg CH_4_ ha^−1^ yr^−1^) was derived from Kang *et al*.^49^, who carried out field measurements on CH_4_ emissions from irrigated paddy fields during rice growing periods in Guangzhou, capital of Guangdong Province. The value of *C*_*CO2-C*_ (8.65 Mg CO_2_ ha^−1^ yr^−1^) is the average value based on two related studies conducted by Liu *et al*.^50^ and Wang *et al*.^51^, with CO_2_ emissions during non-growing season measured by static chamber-gas chromatography and eddy covariance technique, respectively. According to the Mass Balance Method, CH_4_ and CO_2_ is transformed into carbon by multiplying the constant of 12/16 and 12/44, respectively.

Furthermore, according to the existing research^52–54^, indirect carbon emissions from cropland utilisation mainly included (1) carbon emissions during the production process of chemical substances, including chemical fertilizers, pesticides and agricultural plastic film; (2) carbon emissions from energy consumption during agricultural machinery usage and cropland irrigation; and (3) organic carbon loss generated by cropland tillage practices. According to IPCC^48^, the total indirect carbon emissions from agricultural activities were calculated through formula below (Eq. (3)):

3 $$C{E}_{crop \mbox{-} indir}=\sum _{i=1}^{n}{E}_{i}=\sum _{i=1}^{n}{T}_{i}\cdot {\delta }_{i}$$

where *CE*_*crop-indir*_ indicates the total indirect carbon emissions from agricultural activities; *E*_*i*_ is the carbon emissions from the *i*-th agricultural carbon source; *T*_*i*_ denotes the consumption (amount) of the fertilizer, pesticide, agricultural plastic film, total power of agricultural machinery, effective irrigation area and sown area of farm crops, with *δ*_*i*_ as the corresponding carbon emission factor. For details of agricultural carbon sources and their corresponding values of carbon emission factors, please see Supplementary Table S5.

Thus, the formula for calculating total carbon emissions from cropland utilisation is shown as follows (Eq. (4)):

4 $$C{E}_{crop}=C{E}_{crop \mbox{-} dir}+C{E}_{crop \mbox{-} indir}$$

where *CE*_*crop*_ represents the total carbon emissions from cropland utilisation; *CE*_*crop-dir*_ and *CE*_*crop-indir*_ denote the direct and indirect carbon emissions produced by cropland utilisation, respectively.

### Carbon emissions estimation from built-up land construction

As the main land-use type supporting human life and production, built-up land consumes a great amount of energy and produces a large quantity of carbon emissions, including from production and delivery of construction materials and installing and constructing buildings^55^. According to Chuai *et al*.^25^, carbon emissions from built-up land construction were estimated by direct energy consumption during building construction phase. We used the following calculation method for measuring carbon emissions produced by the construction of built-up land (Eq. (5)).

5 $$C{E}_{built \mbox{-} up}=\sum _{i=1}^{n}{C}_{energy \mbox{-} i}=\sum _{i=1}^{n}{Q}_{energy \mbox{-} i}\times {K}_{energy \mbox{-} i}\times ({V}_{C{O}_{2} \mbox{-} i}+{V}_{C{H}_{4} \mbox{-} i})$$

where *CE*_*built-up*_ refers to annual total carbon emissions during the construction phase of built-up land; *C*_*energy-i*_ represents the quantity of carbon emissions from energy *i*; *Q*_*energy-i*_ is the quantity of energy consumption by energy *i*; *K*_*energy-i*_ is the per unit calorific value of energy *i*; and *V*_*CO2-i*_ and *V*_*CH4-i*_ are the carbon emission factors for CO_2_ and CH_4_ from energy *i*, respectively. As noted, since China’s energy carbon emission factors are still under study and we are still lacking published standards for the factors in China, in this study we mainly used emission factors from IPCC^25^, which were detailedly described in Supplementary Table S6.

Given that energy consumption was also involved in the calculation of indirect carbon emissions from cropland utilisation. Thus, to avoid overestimation of carbon emissions from the construction of built-up land, energy consumption during agricultural machinery usage and cropland irrigation was subtracted from the consumption of the corresponding energy type when calculating carbon emissions related to the built-up land construction.

Total carbon emissions of land use were calculated as the sum of carbon emissions from cropland utilisation and the construction of built-up land (Eq. (6)).

6 $$C{E}_{Total}=C{E}_{crop}+C{E}_{built \mbox{-} up}$$

where *CE*_*Total*_ is the total carbon emissions of land use; *CE*_*crop*_ is carbon emissions from cropland utilisation; *CE*_*built-up*_ is carbon emissions from the construction of built-up land. It is worth noting that Eqs (1–6) was applied on multiple spatial scales, including the provincial, sub-provincial and city scale.

### Carbon emissions intensity calculation

Three forms of carbon emissions intensity (CEI) were calculated and analysed in this study. CEI per unit land area provides a measure of the carbon emissions allocated to a unit of terrestrial land. CEI per unit GDP provides a measure of the carbon emissions required to produce a unit of economic activity^56^. Meanwhile, per capita carbon emissions reflects carbon emissions intensity based on population. Here, three forms of CEI were calculated by the following methods (Eqs (7–9)).

7 $$CE{I}_{land}=\frac{C{E}_{Total}}{L}$$

8 $$CE{I}_{gdp}=\frac{C{E}_{Total}}{G}$$

9 $$CE{I}_{population}=\frac{C{E}_{Total}}{P}$$

where *CEI*_*land*_, *CEI*_*gdp*_ and *CEI*_*population*_ represent CEI per unit land area, CEI per unit GDP and per capita carbon emissions, respectively. *CE*_*Total*_ is annual total carbon emissions of land use; *L*, *G*, *P* denotes annual total land area, annual gross domestic product and annual permanent resident population, respectively.

### Statistical analyses

In this study, Pearson correlation coefficient (*r*) was used to investigate how social-economic development influenced the quantity and direction of carbon emissions on the provincial and city scale. We used regression analysis to explore whether the relationship between economic growth and CEI was consistent with the environmental Kuznets curve (EKC) hypothesis (i.e. inverted U-shaped curve)^57^. The formula used is as follows (Eq. (10)):

10 $$y=ax+b{x}^{2}+c$$

where *y* represents the CEI per unit GDP; *x* denotes GDP per capita (the intuitive indication of economic growth); *a*, *b*, and *c* are the equation coefficients, which determine the curve relationship between economic growth and CEI. Specifically, if the coefficient of x in the quadratic equation of one unknown is a positive value and the coefficient of x^2^ is negative, an inverted U-shaped curve relationship is thus formed^38^. All statistical analyses in this study were performed using the SPSS software package (version 16.0). Statistically significant differences were set with P values = 0.05.

## Results

### Land use/land cover area and changes

Our study found that different land use/land cover types in Guangdong experienced varying degrees of change in area from 2005 to 2013, primarily presented as increasing of built-up land and water bodies, and decreasing of grassland, forest land, cropland and barren land (Supplementary Table S3 and Fig. S1). Field surveys showed that the overall interpretation accuracy of first-level classification was more than 90%. Land use transition analysis (Table 1) shows that a total of 12569.46 km^2^ of land across Guangdong Province changed in terms of its land type, which accounted for 7.14% of the entire study area. As the largest land cover type in Guangdong, forest land showed a decreasing trend over the study period, with a declining rate of 1.61%. The area transferred out of forest land was 4321.19 km^2^, mainly occupied by cropland and built-up land. Guangdong was also rich in cropland, which had a much lower change rate (−0.39%). A total of 2096.09 km^2^ cropland was converted to built-up land, accounting for 51.63% of the total area transferred out of cropland. Forest land was the second type of land cover to occupy cropland, with the area of 1224.15 km^2^. Grassland continually decreased between 2005 and 2013, at a rate of 13.66% and area of 1218.46 km^2^. A total of 932.48 km^2^ grassland was converted to forest land. Built-up land underwent the largest area change (2872.06 km^2^), with an increase rate of 23.19%. The transfer-out of built-up land was mainly occupied by cropland, comprising 1008.50 km^2^, which accounted for 66.46% of the entire transfer-out area. However, the transfer-out area of built-up land to cropland was only half of the transfer-out area of cropland to built-up land. Water bodies accounted for approximately 5% of the total area of the province, with a rate of increase of 1.91% from 2005–2013. Water bodies were mainly converted to built-up land and cropland, with 492.00 km^2^ and 288.20 km^2^ transferred, respectively. Although barren land accounted for less than 1% of the total land area of Guangdong, it had the greatest rate of change in area (48.23%). Barren land was mainly transferred to forest land and built-up land.

**Table 1:** Land use transition matrix of Guangdong Province between 2005 and 2013 (km^2^).

2013	Cropland	Forest land	Grassland	Built-up land	Water bodies	Barren land	Total
2005							
Cropland	**36058**.**99**	1224.15	94.32	2096.09	642.74	2.81	40119.10
Forest land	2317.95	**101744**.**08**	228.42	1510.40	226.51	37.92	106065.27
Grassland	291.23	932.48	**7351**.**20**	281.08	49.14	13.99	8919.12
Built-up land	1008.50	267.87	16.72	**10836**.**71**	222.71	1.60	12354.11
Water bodies	288.20	136.10	11.12	492.00	**7279**.**36**	0.51	8207.28
Barren land	12.07	99.62	0.88	35.08	27.27	**123**.**40**	298.33
Total	39976.94	104404.30	7702.66	15251.36	8447.74	**180**.**22**	**175963**.**21**

### Vegetation carbon sinks and carbon emissions estimation

**Figure 2:**
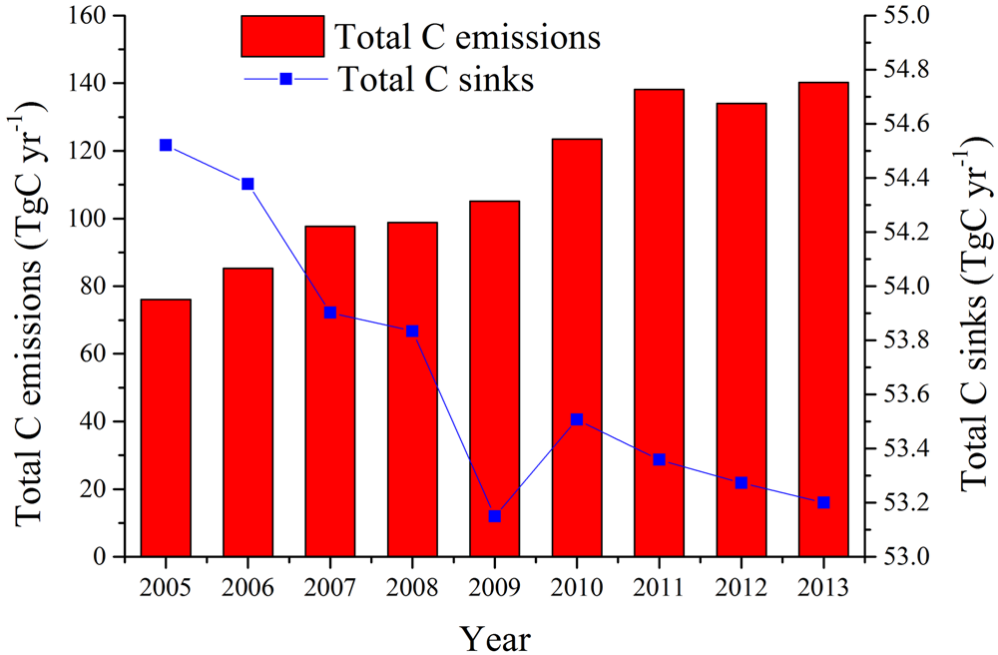
Temporal changes of provincial carbon emissions and vegetation carbon sinks from 2005–2013. Bars represent annual quantity of carbon emissions on new built-up land and cropland. Line represents annual carbon sinks contributed by forest land and grassland.

On a provincial scale, vegetation carbon sinks from both forest land and grassland did not change dramatically from 2005–2013, only decreasing from 54.52 to 53.20 TgC yr^−1^ (Fig. 2 and Table 2). Forest land contributed over 93% to annual vegetation carbon sinks, serving as the most important carbon sink type in Guangdong Province. Meanwhile, vegetation carbon sinks from grassland attained 31.36 TgC in total over the nine years. Provincial carbon emissions on both new built-up land and cropland sharply increased from 76.11 to 140.19 TgC yr^−1^ between 2005–2013, with an average annual rate of increase of 10.52% (Fig. 2 and Table 2). The cumulative carbon emissions in Guangdong were 999.07 TgC from 2005–2013, which corresponded to 2.07 times the total carbon sinks over the study period. The ratio of carbon emissions to carbon sinks kept increasing from 2005–2013, reaching 2.64 in 2013. Carbon emissions from the construction of built-up land increased from 63.74 to 127.49 TgC yr^−1^, an increase of 1.0 times. Built-up land construction contributed 83.75–90.94% to annual provincial carbon emissions, serving as the greatest contributor to carbon emissions in Guangdong. In contrast, carbon emissions from cropland utilisation only increased by 0.026 times, from 12.37 to 12.70 TgC yr^−1^.

**Table 2:** Calculation results of carbon emissions, vegetation carbon sinks, and carbon emissions intensity (CEI) on the provincial scale from 2005–2013.

Year	C emissions on new built-up land (TgC yr^−1^)	C emissions on cropland (TgC yr^−1^)	C sink from forest land (TgC yr^−1^)	C sink from grassland (TgC yr^−1^)	Total C emissions (TgC yr^−1^)	Total C sinks (TgC yr^−1^)	Ratio of total C emissions to sinks	Net C emissions (TgC yr^−1^)	CEI per unit land area (MgC yr^−1^ ha^−1^)	CEI per unit GDP (MgC yr^−1^ 10^−4^ USD)	Per capita C emissions (MgC yr^−1^)
2005	63.74	12.37	50.83	3.69	76.11	54.52	1.40	21.59	4.32	2.76	0.83
2006	73.33	11.96	50.71	3.67	85.29	54.38	1.57	30.91	4.84	2.56	0.92
2007	85.63	12.11	50.24	3.67	97.74	53.90	1.81	43.84	5.55	2.34	1.03
2008	86.61	12.22	50.18	3.66	98.83	53.83	1.84	45.00	5.62	1.87	1.04
2009	92.78	12.40	49.53	3.62	105.17	53.15	1.98	52.02	5.98	1.82	1.04
2010	111.01	12.48	50.12	3.39	123.49	53.51	2.31	69.99	7.01	1.82	1.18
2011	125.56	12.64	50.10	3.26	138.20	53.36	2.59	84.84	7.84	1.68	1.32
2012	121.29	12.76	50.06	3.22	134.05	53.27	2.52	80.77	7.62	1.48	1.27
2013	127.49	12.70	50.01	3.19	140.19	53.20	2.64	86.99	7.96	1.40	1.32

**Figure 3:**
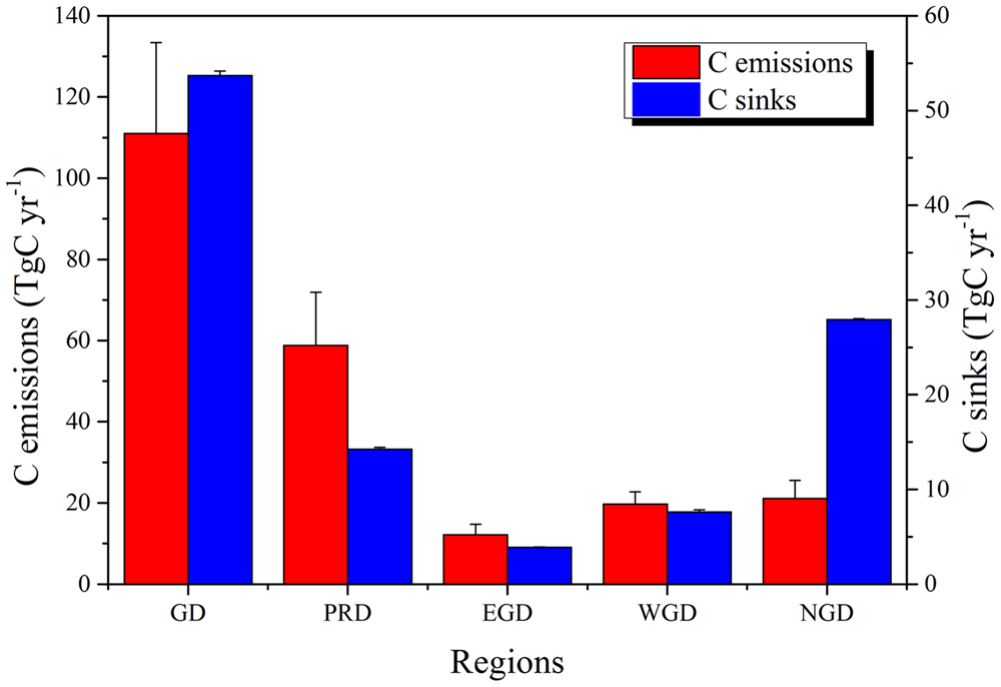
The mean and standard deviations of carbon emissions and vegetation carbon sinks of different regions in Guangdong Province between 2005 and 2013. The red and blue bars represent the mean value of carbon emissions and carbon sinks, respectively, with vertical bars representing + one standard deviation. GD: Guangdong Province; PRD: Pearl River Delta; EGD: East Guangdong; WGD: West Guangdong; NGD: North Guangdong.

**Figure 4:**
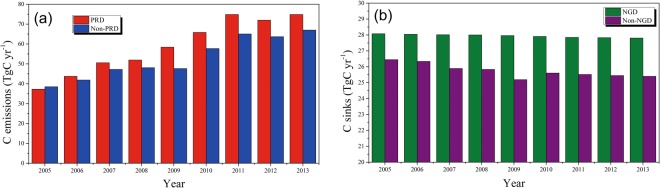
Regional comparisons of carbon emissions and vegetation carbon sinks. (**a**) Annual carbon emissions in Pearl River Delta (PRD) and non-PRD regions (i.e. East Guangdong, West Guangdong and North Guangdong). The red and blue bars represent carbon emissions of PRD and non-PRD, respectively. (**b**) Annual vegetation carbon sinks in North Guangdong (NGD) and non-NGD (i.e. Pearl River Delta, East Guangdong, and West Guangdong). The green and purple bars represent carbon sinks of NGD and non-NGD, respectively.

On a sub-provincial scale, annual carbon emissions of each of the four regions presented a generally increasing trend, of varying degrees, from 2005–2013. Vegetation carbon sinks of these regions also showed a generally decreasing trend (Supplementary Fig. S2). Nevertheless, in terms of absolute values, carbon emissions and carbon sinks in Guangdong showed typical regional differences (Fig. 3). The mean and standard deviations of carbon emissions in Pearl River Delta during 2005–2013 was 58.82 ± 13.09 TgC yr^−1^, higher than other three regions combined (Fig. 4a). This was followed by North Guangdong (21.10 ± 4.42 TgC yr^−1^), West Guangdong (19.70 ± 3.02 TgC yr^−1^), and East Guangdong (12.17 ± 2.56 TgC yr^−1^), which had only a fifth of that of Pearl River Delta. Meanwhile, the mean and standard deviations of vegetation carbon sinks in North Guangdong during 2005–2013 was 27.94 ± 0.09 TgC yr^−1^, larger than other three regions combined (Fig. 4b), followed by Pearl River Delta (14.23 ± 0.22 TgC yr^−1^), West Guangdong (7.62 ± 0.23 TgC yr^−1^), and East Guangdong (3.89 ± 0.04 TgC yr^−1^), accounting for only 14% of that of North Guangdong.

At the city scale, carbon emissions underwent more drastic spatial and temporal changes than vegetation carbon sinks during the study period (Supplementary Figs S3–S6). For further analysis, we took the year 2013 as an example (Table 3). Cities were divided into high-intensity, moderate-intensity, and low-intensity carbon emissions areas. Guangzhou, Shenzhen, Foshan, Dongguan, Huizhou, Jiangmen, Zhanjiang, Maoming and Qingyuan were high-intensity because their carbon emissions were higher than the average provincial level (6.68 TgC yr^−1^). Zhongshan, Zhaoqing, Jieyang, and Meizhou were placed into the moderate-intensity class. Zhuhai, Shantou, Chaozhou, Shanwei, Yangjiang, Shaoguan, Heyuan, and Yunfu were low-intensity. Guangzhou, the provincial capital, had the highest amount of carbon emissions among the 21 cities (13.51 TgC yr^−1^). Meanwhile, in terms of carbon sinks, Shaoguan produced the largest carbon sink in 2013 compared with other 20 cities (6.76 TgC yr^−1^), followed by Qingyuan, Meizhou, Heyuan and Zhaoqing, all much higher than the average level of the whole province (2.53 TgC yr^−1^). With regard to the ratio of total emissions to total sinks, Shaoguan, Heyuan and Meizhou attained the values less than 1, indicating that they were the only three cities playing the role of net carbon sink in 2013. Meanwhile, Dongguan had the highest ratio of total emissions to total sinks (32.65), over 12 times the average provincial level (2.64).

**Table 3:** Calculation results of carbon emissions, vegetation carbon sinks, and carbon emissions intensity (CEI) on the city scale in 2013.

City	C emissions on new built-up land (TgC yr^−1^)	C emissions on cropland (TgC yr^−1^)	C sinks from forest land (TgC yr^−1^)	C sinks from grassland (TgC yr^−1^)	Total C emissions (TgC yr^−1^)	Total C sinks (TgC yr^−1^)	Ratio of total C emissions to sinks	Net C emissions (TgC yr^−1^)	CEI per unit land area (MgC yr^−1^ ha^−1^)	CEI per unit GDP (MgC yr^−1^ 10^−4^ USD)	Per capita C emissions (MgC yr^−1^)
Guangzhou	13.01	0.50	1.43	0.05	13.51	1.48	9.12	12.03	19.01	0.54	1.05
Shenzhen	8.22	0.02	0.31	0.02	8.24	0.33	25.05	7.91	44.90	0.35	0.78
Foshan	11.02	0.15	0.38	0.01	11.17	0.39	28.39	10.77	28.77	0.99	1.53
Zhuhai	3.08	0.05	0.22	0.00	3.13	0.22	14.09	2.91	22.07	1.17	1.97
Dongguan	10.07	0.06	0.30	0.01	10.13	0.31	32.65	9.82	42.32	1.14	1.22
Zhongshan	4.94	0.07	0.16	0.00	5.01	0.16	30.75	4.85	30.50	1.18	1.58
Huizhou	8.72	0.63	3.46	0.15	9.35	3.61	2.59	5.75	8.28	2.16	1.99
Jiangmen	7.54	0.96	2.12	0.15	8.50	2.27	3.75	6.23	9.22	2.63	1.89
Zhaoqing	4.88	0.90	5.30	0.09	5.79	5.39	1.07	0.40	3.90	2.16	1.44
Shantou	4.20	0.25	0.26	0.07	4.45	0.33	13.48	4.12	21.64	1.76	0.81
Chaozhou	2.55	0.22	0.68	0.19	2.77	0.87	3.17	1.90	8.98	2.20	1.02
Jieyang	4.53	0.51	1.14	0.20	5.04	1.34	3.77	3.70	9.69	1.94	0.84
Shanwei	2.94	0.44	1.02	0.28	3.38	1.30	2.61	2.08	7.10	3.11	1.13
Zhanjiang	10.08	1.71	1.84	0.01	11.79	1.85	6.36	9.94	9.90	3.55	1.65
Maoming	6.38	1.11	3.34	0.13	7.49	3.47	2.16	4.03	6.63	2.15	1.25
Yangjiang	4.01	0.76	1.98	0.10	4.77	2.08	2.30	2.69	6.21	2.84	1.92
Shaoguan	3.98	0.95	6.42	0.34	4.93	6.76	0.73	−1.83	2.70	3.02	1.70
Meizhou	4.89	0.89	5.60	0.47	5.78	6.07	0.95	−0.28	3.66	4.48	1.34
Qingyuan	7.12	1.06	6.09	0.47	8.18	6.56	1.25	1.62	4.33	4.64	2.16
Heyuan	3.44	0.84	5.50	0.30	4.28	5.80	0.74	−1.52	2.74	3.89	1.41
Yunfu	3.56	0.56	2.45	0.17	4.12	2.62	1.57	1.50	5.30	4.24	1.70

It is worth noting that “City” in this study is second only to the administrative division of the province.

### Relationship between carbon emissions and social-economic development

**Figure 5:**
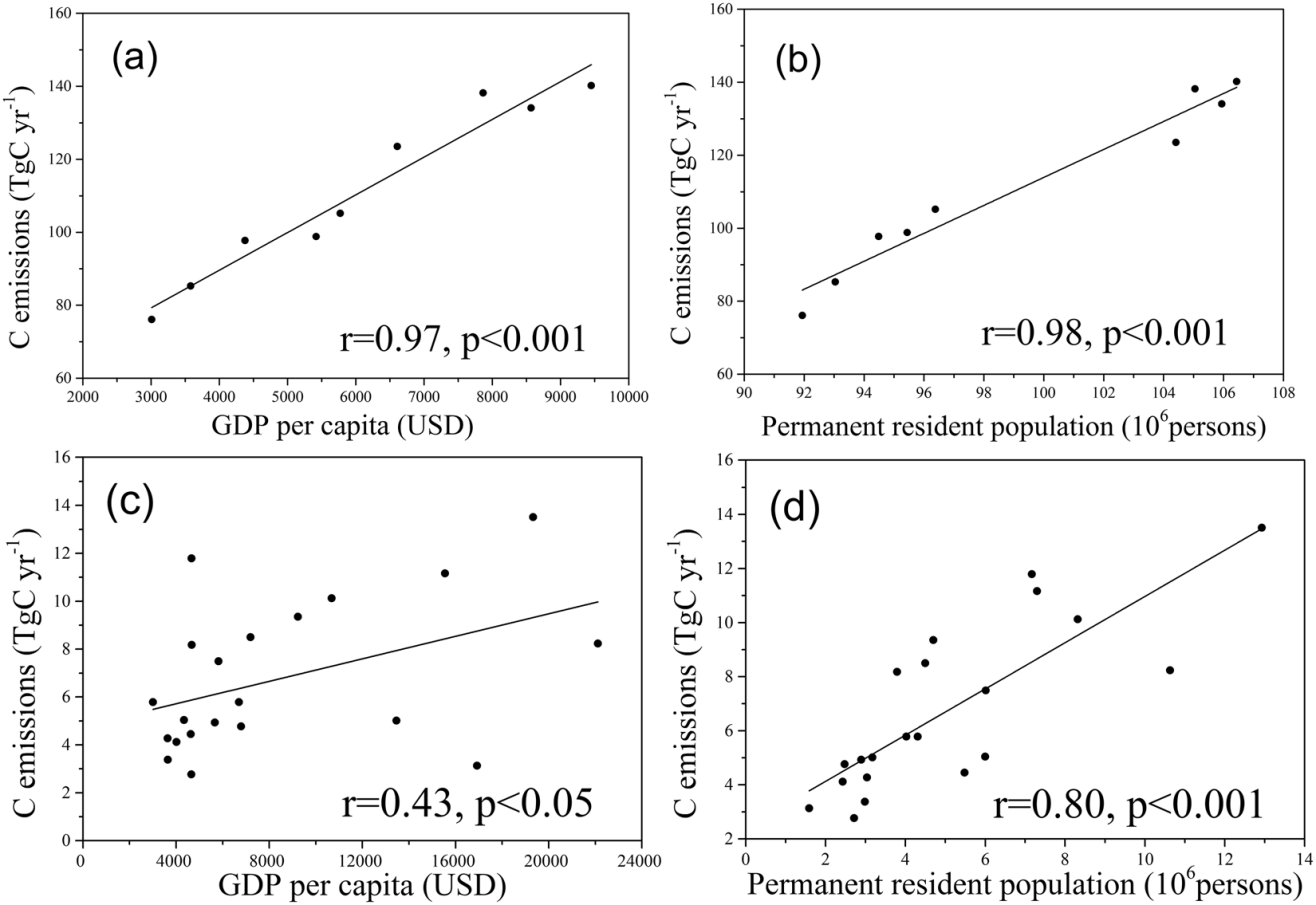
Results of correlation analysis between carbon emissions and social-economic factors. (**a**) Correlation between GDP per capita and annual carbon emissions at the provincial level. (**b**) Correlation between permanent resident population and annual carbon emissions at the provincial level. (**c**) Correlation between GDP per capita and carbon emissions at the city level in 2013. (**d**) Correlation between permanent resident population and carbon emissions at the city level in 2013. Two-tailed test of significance was used in the analysis.

Correlation analysis was conducted to quantitatively evaluate the relationship between social-economic factors and carbon emissions on a provincial and city scale (Fig. 5). GDP per capita had a significant, positive correlation with annual carbon emissions at the provincial level (r = 0.97, p < 0.001) (Fig. 5a). There was also a significant positive correlation between permanent resident population and annual carbon emissions, with a higher Pearson’s correlation coefficient (r = 0.98, p < 0.001) (Fig. 5b), suggesting that population growth promotes energy demand and thus increases carbon emissions at the provincial level. Likewise, taking 2013 as an example on the city scale, GDP per capita had a positive impact on carbon emissions, (r = 0.43, p < 0.05), relatively lower than that of the province (Fig. 5c). Meanwhile, permanent resident population also had a highly positive correlation with city-produced carbon emissions (r = 0.80, p < 0.001) (Fig. 5d), although the correlation coefficient was comparatively lower than that of the province. Results of this analysis indicate that social-economic development is the main driver of increasing carbon emissions.

### Spatiotemporal changes of carbon emissions intensity

**Figure 6:**
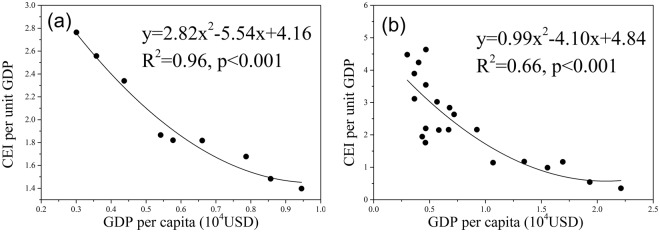
Results of regression analysis between economic growth and carbon emissions intensity. (**a**) Fitted curve between GDP per capita and CEI per unit GDP on the provincial scale from 2005–2013; (**b**) Fitted curve between GDP per capita and CEI per unit GDP on the city scale in 2013. The unit of Y-axis in (**a**) and (**b**) is MgC yr^−1^ 10^−4^ USD.

On the provincial scale, CEI per unit land area displayed an increasing trend, rising from 4.32 to 7.96 MgC yr^−1^ ha^−1^ from 2005–2013 (Table 2), representing an increase of 10.55% per year. In contrast, CEI per unit GDP declined from 2.76 to 1.40 MgC yr^−1^ 10^−4^ USD, i.e. a reduction of 6.18% per year. The relationship between GDP per capita and CEI per unit GDP formed a half-U shape curve (Fig. 6a), which means that carbon emission efficiency in Guangdong Province improved with economic growth. Per capita carbon emissions displayed a generally increasing trend, rising from 0.83 to 1.32 MgC yr^−1^, with an average annual growth rate of 7.39%.

On the city scale, we focused on the spatial differences among 21 cities in terms of CEIs (Table 3). For CEI per unit land area, seven cities, mostly in PRD, far exceeded the provincial average value in 2013 (7.96 MgC yr^−1^ ha^−1^). Shenzhen had the largest value (44.90 MgC yr^−1^ ha^−1^) among the 21 cities, nearly 16 times higher than the lowest value (Shaoguan). In contrast, there were eight cities, mostly in North Guangdong, with a significantly lower value than the provincial average.

For CEI per unit GDP, Guangzhou, Shenzhen, Foshan, Zhuhai, Dongguan, and Zhongshan, all in PRD, had values lower than the average level of the province (1.40 MgC yr^−1^ 10^−4^ USD). Therefore, these cities could be categorized as having high-efficiency carbon emissions. The remaining 15 cities could thus be categorized as low-efficiency carbon emitters. Although total carbon emissions in Guangzhou and Foshan were among the highest in the province, their carbon emission efficiency was also among the highest (0.54 and 0.99 MgC yr^−1^ 10^−4^ USD in Guangzhou and Foshan, respectively). The relationship between GDP per capita and CEI per unit GDP was also a half-U shape (Fig. 6b), suggesting that with the rapid economic development of a city, carbon emission efficiency also constantly increases.

For per capita carbon emissions, there were 13 cities with values higher than the provincial average (1.32 MgC yr^−1^), and eight cities with values lower than the average. Shenzhen had the lowest value among the cities (0.78 MgC yr^−1^), despite having high total carbon emissions in 2013.

## Discussion

### Land use/land cover and carbon emissions estimation

Our study shows that Guangdong Province didn’t undergo drastic LULCC dynamics from 2005 to 2013, with only 7.14% of land area having experienced changes in land types (Table 1), which is much lower than that in Jiangsu Province^26^. Built-up land has been continuously expanded from 2005–2013, mainly at the expense of cropland and forest land. As the most populous and affluent province in China^29^, rapid urbanization and high-speed economic development are the main drivers of significant built-up area expansion^30,58,59^. In contrast, cropland decreased from 2005 to 2013, primarily due to transformation into built-up land, but its rate of decrease was only 0.39%, which can be mainly attributed to the national farmland protection policy^60^. As one of the major rice cultivation regions in China^46^, Guangdong Province has made great efforts to preserve cropland, especially farmland with great production potential. Similarly, although forest land showed a decreasing trend over the study period, but it owned a low declining rate of 1.61%, which can be explained by the fact that sustainable development of forestry and ecological protection were initiated by the local government in 2005^61,62^. Grassland is the only ecological land cover type undergoing a relatively high degree of decline in area (13.66%), mainly converted to forest land, which increased carbon sink capacity to a certain extent^25^.

Land use carbon emissions account for a large proportion of human-driven carbon emissions^48^. In our study, we defined land as a carrier for carbon emissions^31^ and we also proposed an assumption that carbon emissions on certain land surfaces will increase or decrease in proportion according to land areas and the amount of energy and materials consumed on the land, which may not entirely conform to the reality. Carbon emissions thus vary widely according to the land types and land surface features and functions^31^. Built-up land contributed the most carbon emissions, which is consistent with other studies^63,64^. This is mainly due to the fact that human activities are associated with high carbon emissions, and these activities are always based on built-up land^65,66^. Therefore, built-up land restrictions play a significant role in reducing local carbon emissions^31^. Specifically, the built-up land should be controlled by rigid management of urban expansion and cropland occupation^23^. Meanwhile, cropland contributed to carbon emissions through agricultural production activities and CH_4_ emissions during the flooding period and CO_2_ emissions during the non-flooding period in paddy fields^54^. Among the various land types, forest land has the highest level of biomass sequestered as vegetation, in accordance with previous research^67,68^. Thus, forest land protection is critical not only for ecological conservation but also for the vegetation carbon sink increase^69^. In contrast, grassland contributed the least to vegetation carbon storage increase due to its relatively small capacity for carbon sequestration^25^. In summary, land use carbon emissions intensity should be effectively reduced through land use structure adjustment and optimization, mainly by strictly limiting the excessive growth of built-up land and promoting the energy use efficiency, in the meantime, increasing ecological land area and improving carbon sequestration efficiency^23^. However, taking into account the differences in regional land use, the local governments should take corresponding actions based on the local practical conditions^31^.

### Regional differences in carbon emissions and carbon sinks

In this study, we found that carbon emissions in Guangdong rose sharply from 2005–2013, while carbon sinks did not change dramatically (Fig. 2). Both carbon emissions and carbon sinks displayed significant regional differences (Figs 3 and 4). On the sub-provincial scale, carbon emission capacities clearly differed by region, in accordance with economic status. This proves that economic growth is one of the main drivers of increasing carbon emissions during regional development^70,71^. Pearl River Delta region contributed more than 50% of provincial carbon emissions during the study period, due to its position as an advanced manufacturing and modern service base with global influence. The total carbon emissions presented a rising trend in the Pearl River Delta region from 2005 to 2013 (Supplementary Fig. S2). As a result, although this region has been benefiting from high economic level brought about by rapid urbanization, it is currently confronting a more serious carbon emissions reduction task^25^. Therefore, in the future, carbon reduction work in the Pearl River Delta region should be concentrated on enhancing energy use efficiency, augmenting the use of green energy types and speeding up industrial upgrading^38^. Guangzhou, the capital city of Guangdong Province, has witnessed the high-speed economic development during the past decades and represents a groundbreaking example of China’s early industrialization and urbanization trends^38^. Early economic development usually comes at the cost of environmental benefits^72^. Guangzhou had the largest amount of carbon released in the Pearl River Delta region and the whole province, totalling 102.36 TgC from 2005–2013, with the annual average growth rate of 10.55%. Furthermore, the annual carbon emissions in Guangzhou experienced a relatively slow growth trend between 2010 and 2013 after initially rapid increase, and a sharp decline in annual carbon emissions was detected in 2010. These data indicate that Guangzhou has achieved a certain degree of economic stability and has reasonable control over the carbon emissions associated with urban development^38^. In Guangzhou-Foshan, Guangzhou is the center for promoting the economic development of Foshan^38^, which explains total carbon emissions in Foshan ranked second in the province.

Meanwhile, carbon sequestration capacity also clearly differed by region, but in a different order (Figs 3 and 4), which is consistent with the spatial distribution of forest vegetation carbon stocks in the four economic regions of Guangdong Province^73^. North Guangdong had the highest capacity for carbon sequestration from 2005 to 2013 because the forest land in this region has been well conserved due to historical reasons and thus can provide significant ecological services for the province^74^. However, due to rapid population and economic development in the Pearl River Delta region, since the early 1980s, primitive forests have rapidly declined and were replaced by plantations, which are used to protect and adjust the urban environment, and instead result in a relatively higher level of forest carbon storage increase in this region, only after North Guangdong^74^. Vegetation carbon sinks in West Guangdong and East Guangdong were low, which could be attributed to the large area of forest land occupied by young plantations^37^.

### Relationships between carbon emissions and social-economic factors

Our study found strong linear correlations between social-economic factors and carbon emissions on the provincial and city scales (Fig. 5a–d). Economic growth has a positive effect on carbon emissions, as shown in other related studies^75–77^. Although rapid economic growth led by industrialization and urbanization has remarkably enhanced per capita incomes and living standards, a considerable amount of energy consumption results in a relatively high, increasing trend of carbon emissions in the region^78^. Guangdong Province needs to change the current mode of economic development by adjusting land policies to create a land-saving, environment- and eco-friendly land use system^79^. Moreover, population growth also significantly increases carbon emissions at the provincial and city levels. Guangdong’s permanent resident population increased from 91.94 to 106.44 million persons from 2005–2013^80^. To satisfy the basic living requirements of a growing population, more energy will be consumed to meet industry, electricity, and transportation needs^81^, and thus generate more carbon emissions. Historical data showed that global population growth is synchronized with growth of energy consumption and carbon emissions^82,83^. Therefore, with the increase of permanent resident population, mainly due to many migrant workers entering Guangdong Province from other underdeveloped provinces of China, annual carbon emissions also correspondingly increase.

According to our study, the relationship between GDP per capita and CEI per unit GDP were half-U shape curves (Fig. 6a,b), indicating that carbon emission efficiency in Guangdong Province improved when the economy grew. However, according to the environmental Kuznets curve (EKC) hypothesis, the relationship between economic growth and environmental pollution follows an inverted U-shaped curve, indicating that in the early stages of economic development, the environment deteriorates with economic growth^71^. When the economic development reaches a certain stage, also referred to as the threshold point, environmental degradation is curbed and the situation improves with further economic development^84^. This is because with economic growth, technology improves and the status of economic growth depending on a large number of investments and energy consumption changes. Guangdong’s economic growth has clearly exceeded the threshold; thus, the carbon emissions intensity decreases with the growing economy. Therefore, by selecting a low-carbon development path, the increasing rate of economic development will be higher than that of energy consumption, and carbon emissions intensity will eventually begin to decrease, creating sustainable and healthy economic growth.

### Uncertainties

In this study, we estimated carbon emissions and sinks in economically developed areas of China from a spatial-temporal perspective, taking Guangdong as a case study. However, there existed some uncertainties in carbon estimations here. Our study proposed a carbon emission factor method for indirectly calculating carbon emissions from built-up land construction based on the theory that a large amount of carbon emissions from energy consumption arise during the building construction and demolition phases^85,86^. Given that China’s energy carbon emission factors are still under study and because published standard factors are still lacking, thus in accordance with most other related research conducted in China, our study mainly adopted the emission factors from the IPCC^25^. This calculation method may not accurately reflect the actual conditions in Guangdong Province, but it makes our research comparable with previous research^25^. In the meanwhile, in our study, we didn’t consider carbon emissions from the building operation phase. According to You *et al*.^85^, based on the 50 years of building usage, the carbon emissions from the building operation phase is 5–7 times higher than that of the building construction and construction materials preparation phases. According to our calculation, the amount of carbon emissions on new built-up land was 98.60 ± 23.53 TgC yr^−1^ during 2005–2013 at the provincial level, contributing more than 83% to annual total carbon emissions (Table 2). This proportion is much larger than previous estimates^85,87,88^. Chuai *et al*.^25^ estimated that anthropogenic carbon emissions from the construction sector increased from 39.05 to 1037.21 TgC yr^−1^ in China between 1995 and 2010. Therefore, the carbon emissions estimate from built-up land construction in our study was approximately a tenth of the national counterpart. Our study also estimated carbon emissions from cropland utilisation, with the value of 12.40 ± 0.27 TgC yr^−1^ from 2005–2013 in the province, which is larger than carbon emissions estimation by Lu *et al*.^24^. This is mainly because they didn’t consider the CH_4_ and CO_2_ emissions from rice production. Zhang *et al*.^46^ estimated the CH_4_ emissions from irrigated rice cultivation in China using a CH4MOD model, with the value of 6.62 Tg CH_4_ yr^−1^ from 2005 to 2009, which is 4.38 times the amount of our estimate in Guangdong Province over the same period. However, considering that the total paddy area in China is 15.79 times of that of Guangdong Province^89^, CH_4_ emissions in our study may have been overrated to a certain extent. In this study, we did not consider the effect of soil organic carbon (SOC) because SOC changes may need much more time compared with vegetation^25,26^.

Our study mainly used a carbon sink factor method to estimate carbon sinks from forest land and grassland, assuming that the carbon sink factor in the same land type remained unchanged over time, and without spatial variations considered^90^. Some scholars have pointed out that such literature-based estimate of carbon factors may only represent carbon uptake for a specific time and fail to consider changes caused by environmental impacts (e.g. climate change)^5^. However, in our study, the main environmental factors (i.e. temperature and precipitation) didn’t show significant interannual variations across the whole province during 2005–2013 (Supplementary Fig. S7), indicating that the environmental conditions remained relatively stable during the study period. Moreover, sensitivity analysis^91^ showed that total carbon sinks estimated in this study area were relatively inelastic with respect to the carbon factor value (Supplementary Table S7). In other words, the adjustments to carbon factors associated with forest land and grassland had very minimal impact on the carbon estimations over the study area. Hence, in theory, our method provides a simple and practical method for the calculation of large-scale carbon sinks, which has been widely used across China^23,31^.

In fact, with the maturation and development of forests, the carbon sequestration capacity of forests in Guangdong Province will continue to grow, which leads to a potential huge carbon sink^37,73^. Meanwhile, the increase of forest biomass carbon storage presented a certain degree of spatial heterogeneity throughout the province^37^. The average carbon storage was larger in North Guangdong than in other three regions from 2005 to 2013. Therefore, in the future, additional studies will be needed to produce modified model parameters that reflect the actual situation in Guangdong Province and its four economic regions by conducting the field experiments, which will improve the accuracy of the estimation on carbon sinks^38^. Furthermore, due to data limitation, carbon sinks of logged forests, bamboo forest, farmland protection forest, and afforested trees were not calculated in this study. In addition, shrubland biomass served as a net carbon sink of 0.022 ± 0.01 PgC yr^−1^, accounting for approximately 30% of forest sink in China in the 1980s^42^. However, shrubland in Guangdong Province was not considered because of the extremely scarce information on the carbon balance of this land cover type. Similarly, due to the lack of relevant experimental data, the carbon effect of water bodies was not considered here, although it can be regarded as a weak carbon sink land cover type^69^. In this regard, our estimate of carbon sink in Guangdong Province during 2005–2013 may be underestimated to a certain extent. Moreover, it is worth noting that the overall carbon sink, in addition to vegetation, includes soil^92^; this was excluded because estimates of the soil carbon sink, especially natural soil, are rarely reported in China since data from repeated inventories is lacking^42^.

In addition to the qualitative descriptions on uncertainty of carbon estimation, it is necessary to discuss the uncertainty in a relatively quantitative way through comparison to other related studies. Xu *et al*.^69^ estimated the carbon sinks of forest land and grassland in Pearl River Delta by applying the improved CASA model and photosynthetic reaction equation, ranging from 9.83 TgC yr^−1^ to 10.50 TgC yr^−1^ during 2005–2013, which is lower than our estimate of 14.49 TgC yr^−1^ to 14.17 TgC yr^−1^ over the same period. Few studies have directly estimated the terrestrial carbon sinks over the whole Guangdong Province. However, as noted, net primary productivity (NPP), as an important component of the ecosystem carbon cycle, reflects not only the production capability of plant communities under natural conditions, but also characterizes the carbon source/sink function of ecosystems^93^. In fact, carbon sinks of forests and grasslands gradually increase with the increase of NPP^43^. Therefore, it is reasonable to compare our estimate of carbon sinks in Guangdong Province with related NPP estimates by previous studies. For example, a recent assessment conducted by Pei *et al*.^94^ simulated an average annual vegetation NPP of 94.77 TgC yr^−1^ in Guangdong Province for the period 2000–2009 using a Biome-BGC model, nearly double our estimate during 2005–2013 (53.68 TgC yr^−1^). A large portion of the difference was due to their estimate comprising the NPP of cropland and shrubland, without subtraction of heterotrophic respiration. Using an improved CASA model with MODIS NDVI, meteorological data and land-use map, Luo and Wang^95^ estimated the terrestrial NPP in Guangdong Province was between 103.50–166.80 TgC yr^−1^ during 2001–2007, which is more than twice the amount of our results. A separate analysis of inventory-satellite-based method, ecosystem modeling and atmospheric inversions estimated that the net terrestrial carbon sink was between 18.6–27.4 TgC yr^−1^ in South China (i.e. Guangdong, Guangxi and Hainan Provinces) during the 1980s and 1990s^42^. In this regard, our study likely overestimated the carbon sinks in Guangdong, which may be attributed to the relatively higher carbon sink factors. Additionally, Janssens *et al*.^96^ estimated a net carbon sink between 135–205 TgC yr^−1^ in the terrestrial biosphere of geographic Europe, the equivalent of 7–12% of carbon emitted by anthropogenic sources. By contrast, our study estimated that up to 38–72% of Guangdong’s carbon emissions produced by built-up land construction and cropland utilisation has been removed by carbon sequestration in forests and grassland. This proportion is much larger than Janssens’s estimate. Moreover, Sleeter *et al*.^97^ estimated the terrestrial carbon sinks in the conterminous United States to be 254 TgC yr^−1^ on average between 1973–2010, almost four times larger than our estimate, while the land area of the conterminous United States is 42 times higher than that of Guangdong Province.

## Conclusion

Our study attempted to provide a comprehensive accounting of carbon estimations at multiple spatial scales during 2005–2013, taking Guangdong as an example. We also explored the relationship between carbon emissions and social-economic development to find out the factors influencing the quantity and direction of carbon emissions in Guangdong Province. An interesting phenomenon relating economic growth and carbon emissions intensity was also discussed in this paper. Although there existed considerable uncertainties in carbon emissions/sinks estimation, and we did not discuss all possible factors influencing carbon emissions, our study nevertheless provides a new insight for Guangdong Province to achieve carbon reduction goals and realize low-carbon development. We believe further study is needed to improve the accuracy of estimation on carbon sinks and emissions in this area and to analyse more social-economic factors related to carbon emissions. Additionally, more attention should be paid to the optimal and feasible land use configurations, effective land use and urban planning, for the purpose of combining economic growth and ecological conservation^98^.

## Electronic supplementary material

Supplementary Information
