# Supplementary material for: Spatial-temporal dynamics of carbon emissions and carbon sinks in economically developed areas of China: a case study of Guangdong Province
Source: Sci Rep. 2018 Sep 6;8:13383. doi: 10.1038/s41598-018-31733-7 (PMC6127195; doi:10.1038/s41598-018-31733-7)
Supplement: Supplementary file 1 — Supplementary Information [file 41598_2018_31733_MOESM1_ESM.pdf]

Supplementary Information for Spatial-temporal dynamics of carbon emissions and carbon sinks in economically developed areas of China: a case study of Guangdong Province

Jie Pei [1,2], Zheng Niu [1,2,*], Li Wang [1,3,*], Xiao-Peng Song [4], Ni Huang [1], Jing Geng [2,5], Yan-Bin Wu [3], Hong-Hui Jiang [6]

[1]*The State Key Laboratory of Remote Sensing Science, Institute of Remote Sensing and Digital Earth, Chinese Academy of Sciences, Beijing 100101, P.R.China*

[2]*University of Chinese Academy of Sciences, Beijing 100049, P.R.China*

[3]*College of Management Science and Engineering, Hebei University of Economics and Business, Shijiazhuang 050061,P.R.China*

[4]*Department of Geographical Sciences, University of Maryland, College Park, Maryland, 20742, USA*

[5]*Key Laboratory of Ecosystem Network Observation and Modeling, Institute of Geographic Sciences and Natural Resources Research, Chinese Academy of Sciences, Beijing 100101, China*

[6]*Key Area Planning Construction and Management Bureau of Longgang, Shenzhen, Shenzhen 518116, P.R.China*

Correspondence and requests for materials should be addressed to L.W. (wangli@radi.ac.cn) or Z.N. (niuzheng@radi.ac.cn)

Supplementary Table S1. Description of yearly satellite images acquired from 2005–2013.

Year of image acquired	Satellite name	Sensor name	Spatial resolution(m)	Swath width(km)	Revisit time(d)
2005	CBERS-02	CCD camera	20	113	26
2006	Landsat 5	Thematic Mapper	30	185	16
2007	Landsat 5	Thematic Mapper	30	185	16
2008	CBERS-02B	CCD camera	20	113	26
2009	CBERS-02B	CCD camera	20	113	26
2010	Landsat 5	Thematic Mapper	30	185	16
2011	Landsat 5	Thematic Mapper	30	185	16
2012	ZY-1 02C	Pan/ Multispectral camera	5/10	60	3-5
2013	Landsat 8	Operational Land Imager	30	170×185	16

Supplementary Table S2. National Ecological Remote Sensing Monitoring Land Use/Land Cover Classification System.

First-level classifications	Second-level classifications
Cropland	Paddy field
	Dry cropland
Forest land	Wood land
	Shrub land
	Sparse woodland
	Other woodland
Grassland	Highly covered grassland
	Moderately covered grassland
	Lowly covered grassland
Water bodies	River and canal
	Lake
	Reservoir, pond
	Glaciers and permanent snow
	Shallow
	Beach land
Built-up land	Urban land
	Rural residential land
	Industry and traffic used land
Barren land	Desert
	Gobi
	Saline and alkaline land
	Swampland
	Bare land
	Rock and gravel land
	Other unused land

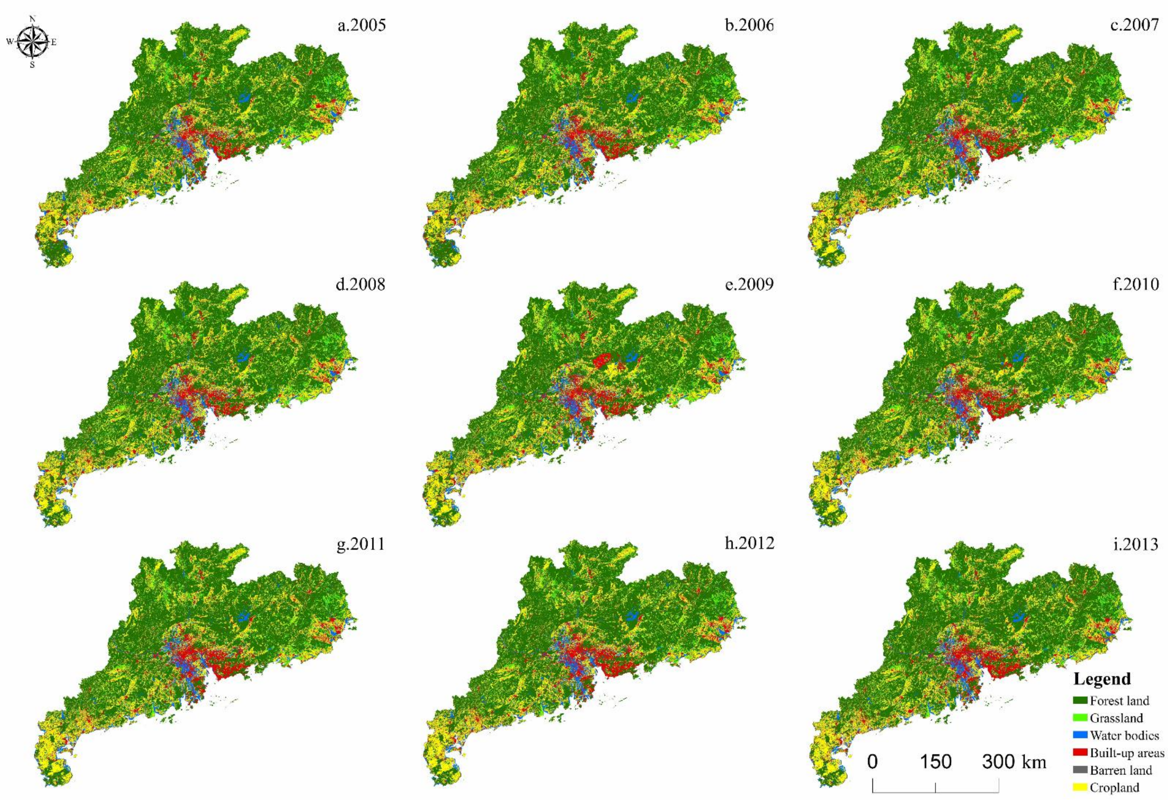

Supplementary Figure S1. The annual land use/land cover map of Guangdong Province from 2005 to 2013, with the first-level classifications of cropland, forest land, grassland, water bodies, built-up land and barren land. Map created using ArcGIS [9.3], (http://www.esri.com/software/arcgis)

Supplementary Table S3. Land use/land cover area and changes in Guangdong Province from 2005–2013 (km^2). Growth rate represents the area increase rate of land types from 2005 to 2013.

land use/land cover types	2005	2006	2007	2008	2009	2010	2011	2012	2013	2005-2013	Growth rate(%)
Cropland	40134.61	39931.99	40848.41	40771.38	41165.59	40731.03	40367.21	40089.42	39979.21	-155.40	-0.39
Forest land	106111.83	105858.20	104877.94	104755.44	103395.28	104633.47	104591.64	104503.40	104408.05	-1703.78	-1.61
Grassland	8921.47	8868.81	8856.62	8829.80	8752.07	8181.28	7872.34	7767.20	7703.01	-1218.46	-13.66
Built-up land	12384.91	13044.00	13052.39	12931.70	13976.07	13892.25	14534.35	14977.51	15256.97	2872.06	23.19
Water bodies	8337.35	8240.43	8280.11	8245.37	8284.88	8468.01	8617.22	8505.84	8496.44	159.09	1.91
Barren land	348.96	313.51	307.58	335.00	409.34	276.72	182.08	180.95	180.64	-168.31	-48.23
**Total**	176239.13	176256.95	176223.04	175868.69	175983.22	176182.77	176164.85	176024.33	176024.32	-214.81	-0.12

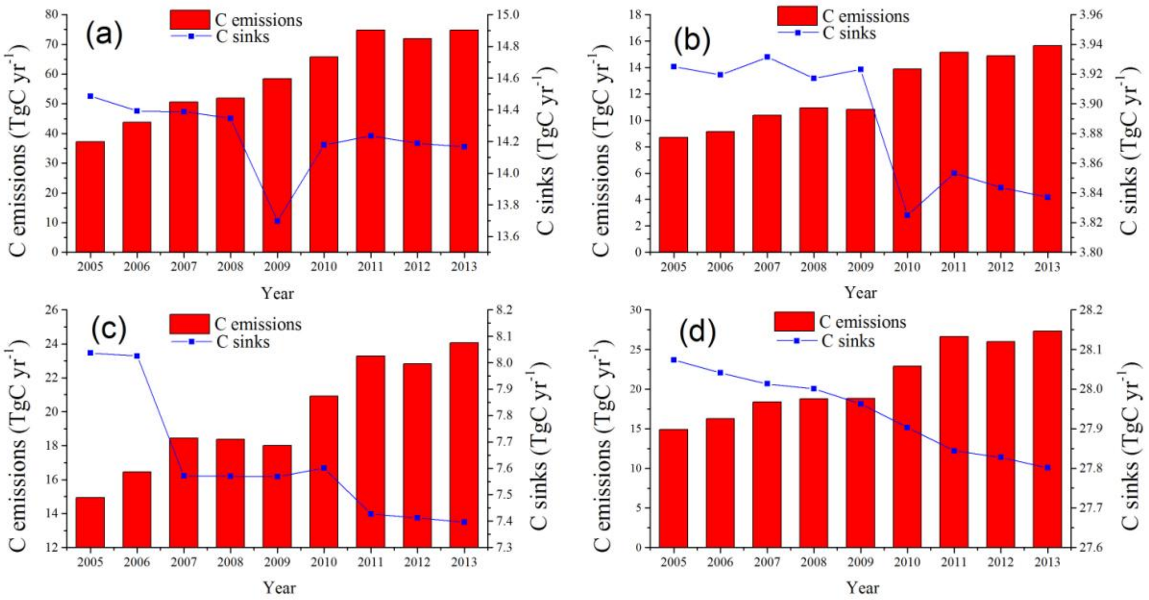

Supplementary Figure S2. Results of estimation on carbon emissions and carbon sinks in (a) Pearl River Delta, (b) East Guangdong, (c) West Guangdong, (d) North Guangdong. Bars represent annual quantity of carbon emissions from the expansion of built-up land and cropland utilisation. Line represents annual carbon sinks contributed by forest land and grassland. At the sub-provincial scale, annual carbon emissions of each of the four regions presented a generally increasing trend, of varying degrees, from 2005–2013. Nevertheless, vegetation carbon sinks of these regions all showed generally decreasing trends.

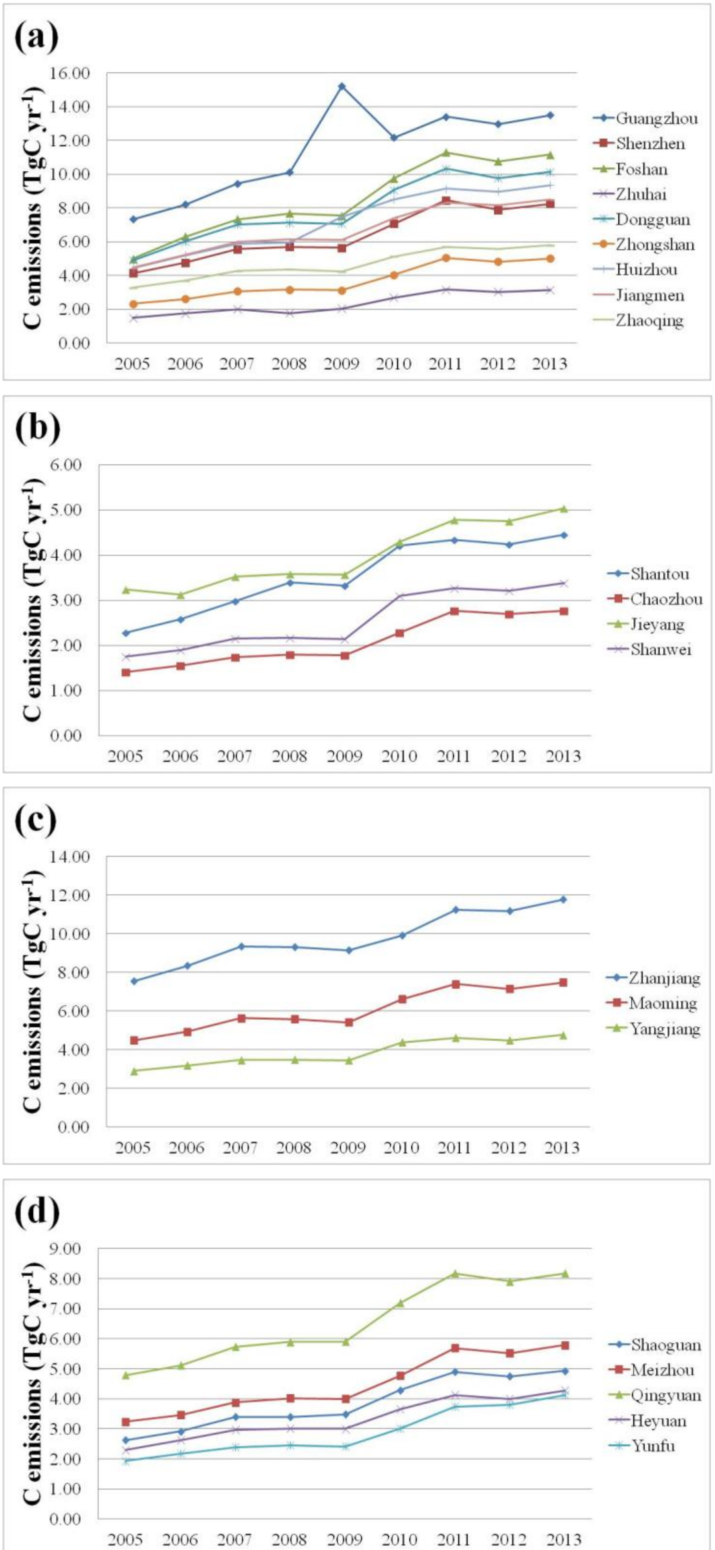

Supplementary Figure S3. Temporal changes of carbon emissions produced by cities in (a) Pearl River Delta, (b) East Guangdong, (c) West Guangdong, (d) North Guangdong.

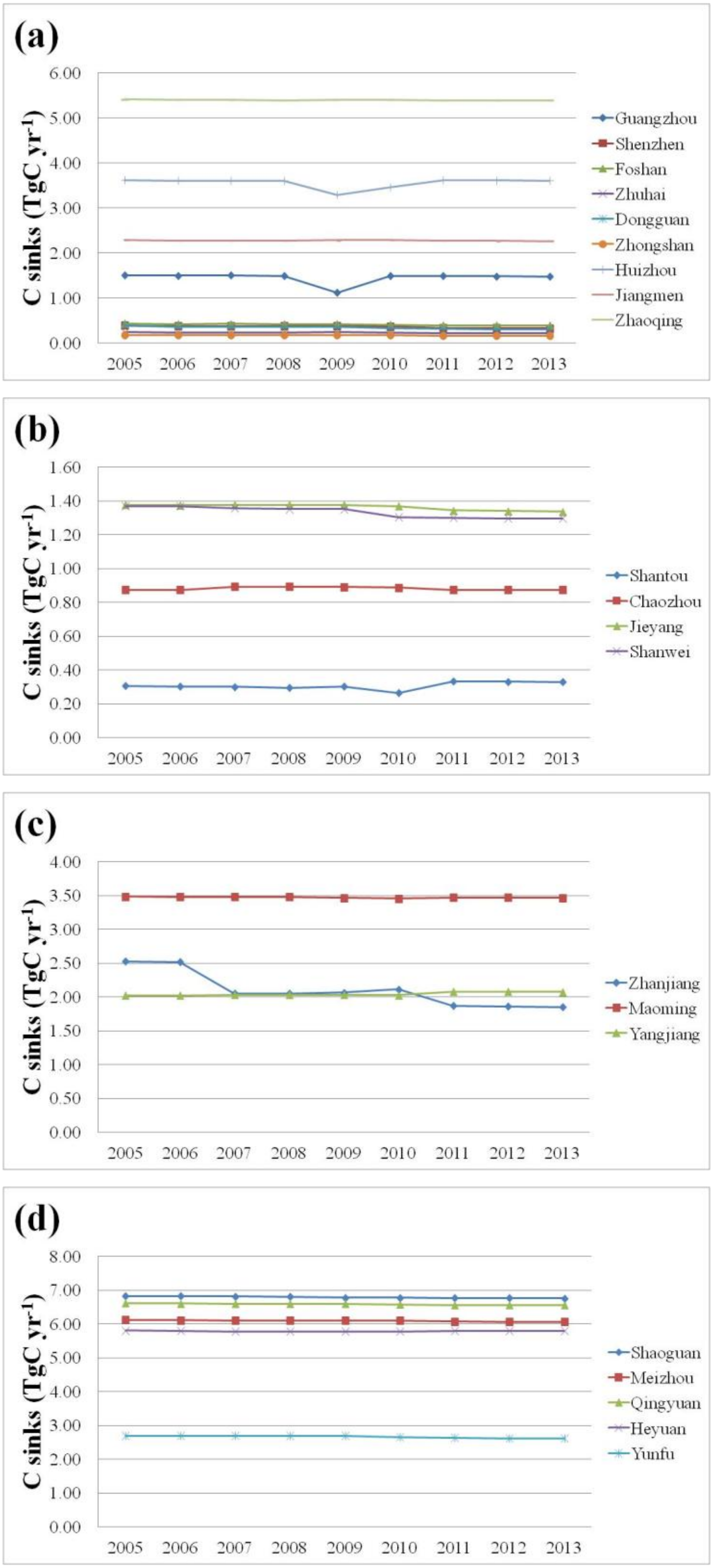

Supplementary Figure S4. Temporal changes of carbon sinks contributed by cities in (a) Pearl River Delta, (b) East Guangdong, (c) West Guangdong, (d) North Guangdong.

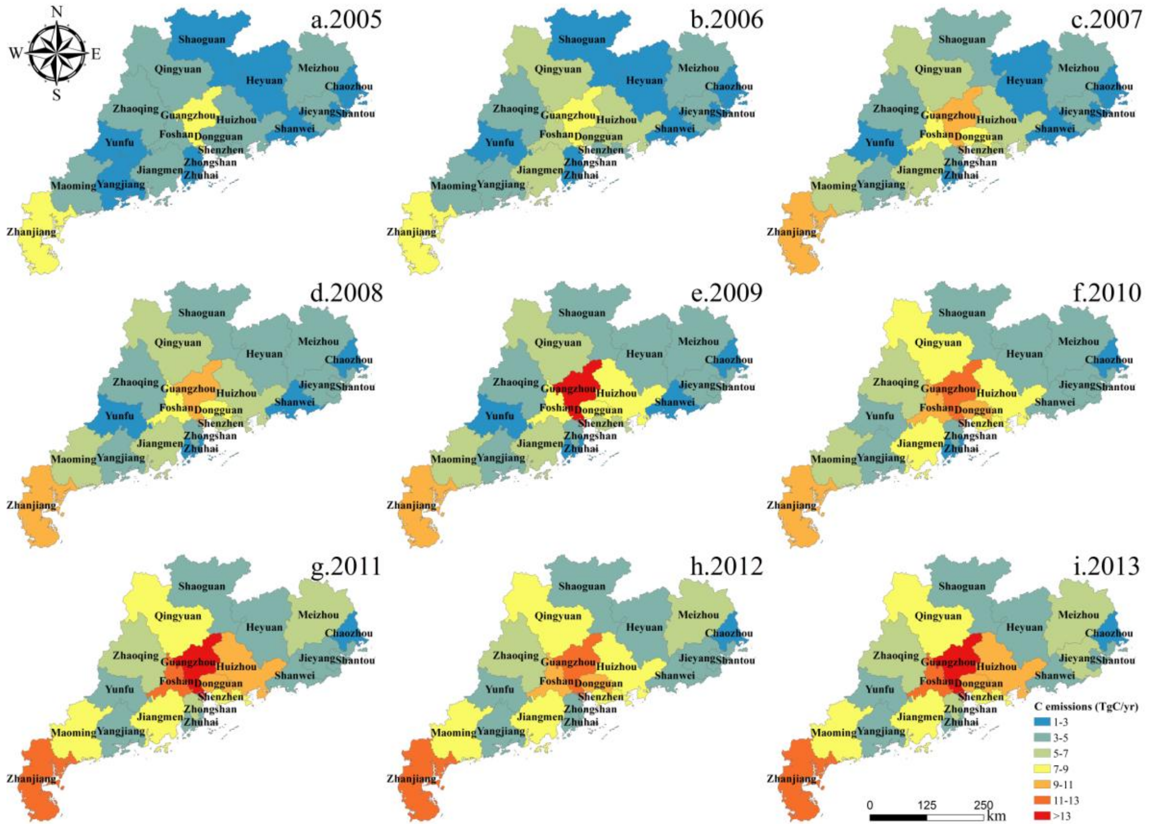

Supplementary Figure S5. Spatial distribution of carbon emissions in 21 cities of Guangdong Province from 2005 to 2013. Note that cities were divided into 7 levels by different coloured representation, i.e. 1-3 TgC yr^{-1}, 3-5 TgC yr^{-1}, 5-7 TgC yr^{-1}, 7-9 TgC yr^{-1}, 9-11 TgC yr^{-1}, 11-13 TgC yr^{-1}, >13 TgC yr^{-1}. Map created using ArcGIS [9.3], (http://www.esri.com/software/arcgis)

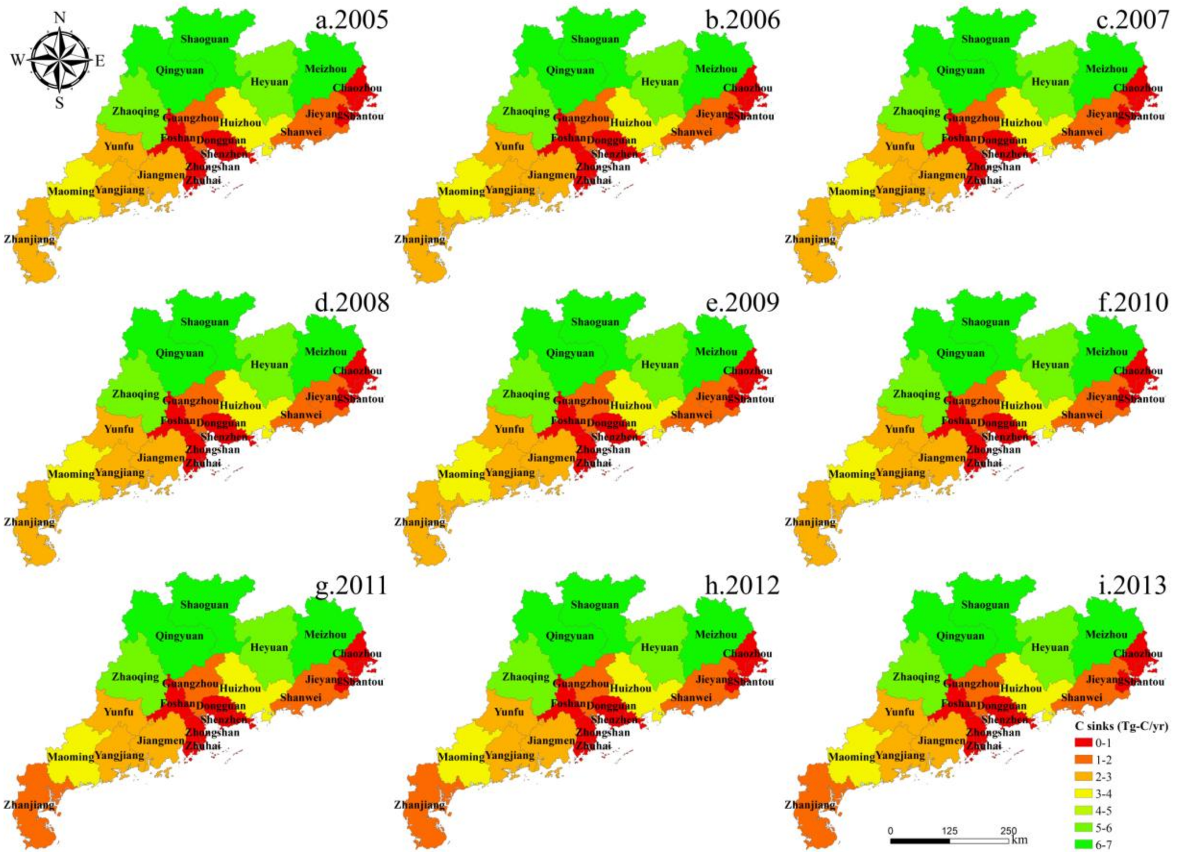

Supplementary Figure S6. Spatial distribution of carbon sinks in 21 cities of Guangdong Province from 2005 to 2013. Note that cities were divided into 7 levels by different coloured representation, i.e. 0-1 TgC yr^{-1}, 1-2 TgC yr^{-1}, 2-3 TgC yr^{-1}, 3-4 TgC yr^{-1}, 4-5 TgC yr^{-1}, 5-6 TgC yr^{-1}, 6-7 TgC yr^{-1}. Map created using ArcGIS [9.3], (http://www.esri.com/software/arcgis)

Supplementary Table S4. Carbon sink factors of different land use/land cover types quoted from relevant literature conducted in Guangdong Province.

Land use/land cover types	Carbon sink factor (MgC yr^{-1} ha^{-1})	Study area	Study period	Calculation method	References
Forest land	4.45	Guangdong Province	1990-2004	Field measurement	[1]
	5.19	Pearl River Delta	2000-2013	CASA model	[2]
	4.73	Pearl River Delta	2003	Field measurement	[3]
**Average**	**4.79**				
Grassland	4.91	Pearl River Delta	2000-2013	CASA model	[2]
	3.37	Guangdong Province	1990-2004	Field measurement	[1]
**Average**	**4.14**				

Supplementary Table S5. Literature-based carbon emission factors of indirect carbon sources of cropland utilisation.

Carbon sources	Carbon emission factor	References
Chemical fertilizer	0.8956 kgC/kg	[4], [5]
Pesticide	4.9341 kgC/kg	[6]
Agricultural plastic film	5.18 kgC/kg	[7]
Agricultural machinery	0.18 kgC/kW	[8]
Agricultural irrigation	266.48 kgC/ha	[9]
Cropland tillage	3.126 kgC/ha	[10]

Supplementary Table S6. Main energy types consumed in Guangdong Province and their corresponding carbon emission factors. Specifically, parameters of per unit calorific value and the carbon emission factors of CH_4 and CO_2 of each energy type were required to calculate the direct energy consumption during building construction phase. Per unit calorific values were mainly obtained from “China Energy Statistical Yearbook” . As for some energy types which were missing in the “China Energy Statistical Yearbook”, per unit calorific values were thus quoted from the Intergovernmental Panel on Climate Change (IPCC) (2006). Carbon emission factors of CO_2 and CH_4 were also obtained from the IPCC (2006). The details are shown below.

**Energy type**	**Per unit calorific value**		**Carbon emission factors of CO_2**	**Carbon emission factors of CH_4**
	**Value**	**Unit**	**（kg C/GJ）**	**(×10^{-3}.kg C/GJ)**
Raw coal	20908	KJ/kg	25.80	0.75
Cleaned coal	26344	KJ/kg	26.21	0.75
Washed coal	9409	KJ/kg	26.95	0.75
Coal products	15910	KJ/kg	26.60	0.75
Briquettes	9409	KJ/kg	26.60	0.75
Coal water slurry	9409	KJ/kg	26.95	0.75
Pulverized coal	9409	KJ/kg	26.95	0.75
Coke	28435	KJ/kg	29.20	0.75
Other coking products	34332	KJ/kg	26.60	2.25
Coke oven gas	17354	KJ/m^3	12.10	0.75
Blast furnace gas	2985	KJ/m^3	70.80	0.75
Other gas	16970	KJ/m^3	60.20	0.75
Natural gas	38931	KJ/m^3	15.30	0.75
Crude oil	41816	KJ/kg	20.00	2.25
Gasoline	43070	KJ/kg	18.90	2.25
Kerosene	43070	KJ/kg	19.60	2.25
Diesel oil	42652	KJ/kg	20.20	2.25
Fuel oil	41816	KJ/kg	21.10	2.25
Liquefied petroleum gas	50179	KJ/kg	17.20	0.75
Refinery gas	46055	KJ/kg	15.70	0.75
Coal tar	33453	KJ/kg	20.00	2.25
Other petroleum products	37681	KJ/kg	20.00	2.25
Electricity	1	KJ/kJ	26.95	0.75
Heat	3596	KJ/KWh	26.95	0.75

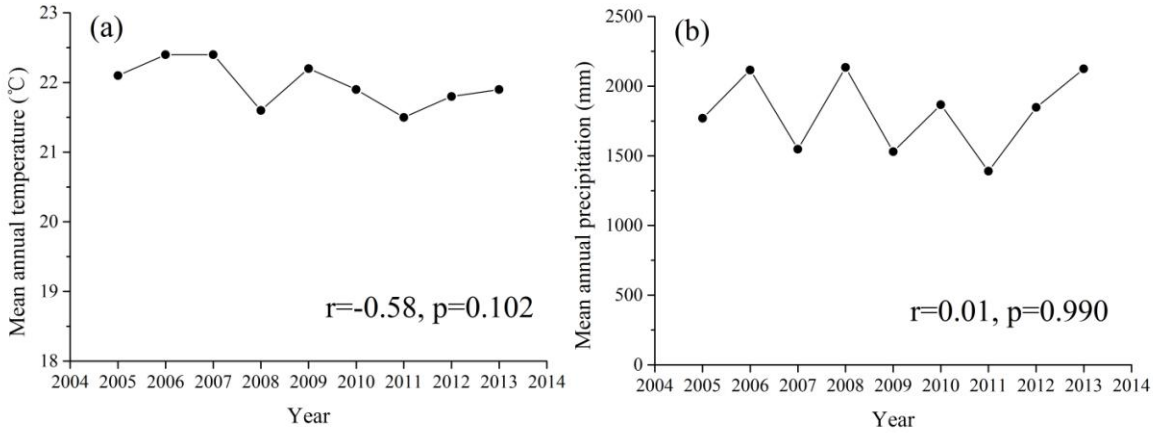

Supplementary Figure S7. Interannual variations of the environmental factors over the whole Guangdong Province from 2005 to 2013. (a) Mean annual temperature variations during 2005–2013, (b) Mean annual precipitation variations during 2005-2013. The meteorological data were obtained from Chinese National Meteorological Information Center (http://data.cma.cn/site/index.html). The results showed that neither of the environmental factors presented significant changes during 2005–2013 in Guangdong Province, indicating that the enviromental conditions remained relatively stable during the study period.

Sensitivity analysis

Considering the carbon emissions from paddy fields and carbon sinks from forest land and grassland were mainly based on empirical coefficients obtained from existing literature, it is thus necessary to introduce an additional sensitivity analysis in order to evaluate their effects on the carbon estimation. It is noted that the carbon emissions from built-up land construction and emissions from indirect carbon sources of cropland (i.e. agricultural machinery, irrigation, tillage, and consumption of materials) are based on energy and material consumption data, and the corresponding carbon coefficients do not subject to environmental impacts. Therefore, these coefficients can be seen as spatially uniform across the whole province, and temporally unchanged. As a result, we didn't involve sensitivity analysis regarding carbon emissions from built-up land contruction and indirect carbon emissions from cropland.

In each analysis, the coefficient of sensitivity (CS) [12] was calculated to quantify the percentage change in the carbon sinks/emissions values for a given percentage change in carbon factor, using the standard economic concept of elasticity [13]. The calculation formula is shown in Equation (S1):

$$CS_{\sin k} = \frac{(Cveg_j - Cveg_i) / Cveg_i}{(Rveg_{jk} - Rveg_{ik}) / Rveg_{ik}} \qquad \text{(S1)}$$

Where CS_{sink} is the CS value of the carbon sink, *Cveg* is the estimated carbon sinks of Guangdong Province, *Rveg* is the carbon sink factor, '*i*' and '*j*' represent the initial and adjusted values, respectively, and '*k*' denotes the land use/land cover type. The same method was also used to calculate the CS value of the carbon emissions from paddy fields. If CS is larger than unity, then the estimated carbon sinks/emissions is considered elastic with respect to that coefficient, while a CS value less than one indicates that the estimated carbon sinks/emissions is inelastic.

In this study, the percentage change of carbon sink/emission factor was set as 50%, and the CS value was calculated using Equation S1 (Supplementary Table S7). Results showed that in all cases, CS was less than unity during 2005–2013, especially the CS value for grassland carbon sink and paddy CH_4 emissions. These results confirmed that the total carbon sinks and total direct carbon emissions from cropland estimated in this study area were relatively inelastic with respect to the carbon factor value. Forest land produced the largest CS value (ca. 0.9323), which can be attribute to the large area of forest land in Guangdong Province. In summary, the sensitivity analysis suggested that the adjustments to carbon factors associated with forest land, grassland and paddy fields had very minimal impact on the carbon estimations over the study area.

Supplementary Table S7. The coefficient of sensitivity (CS) resulting from adjustment of carbon factors (±50%). It is worth noting that because of the area of paddy fields remained unchanged during the flooding and non-flooding period, therefore, the CS values of CO_2 emissions and CH_4 emissions from paddy fields didn't change during 2005–2013.

	**Forest land**	**Grassland**	**Paddy_CO_2**	**Paddy_CH_4**
2005	0.9323	0.0677	0.8055	0.1945
2006	0.9325	0.0675	0.8055	0.1945
2007	0.9320	0.0680	0.8055	0.1945
2008	0.9321	0.0679	0.8055	0.1945
2009	0.9318	0.0682	0.8055	0.1945
2010	0.9367	0.0633	0.8055	0.1945
2011	0.9389	0.0611	0.8055	0.1945
2012	0.9396	0.0604	0.8055	0.1945
2013	0.9401	0.0599	0.8055	0.1945

References

1. Xiao, H. J. The estimation of the terrestrial carbon budget in Guangdong Province and discussion on the mechanism of its change. Master's Thesis. Graduate School of Chinese Academy of Sciences, Guangzhou (2006) (in Chinese).

2. Xu, Q., Dong, Y., & Yang, R. Influence of different geographical factors on carbon sink functions in the Pearl River Delta. *Sci. Rep*. **7(1)**, 110; DOI:10.1038/s41598-017-00158-z (2017).

3. Peng, J. Y. Roles of vegetation on balance of carbon and oxygen in the Pearl River Delta. *Acta Scientiarum Naturalium Universitatis Sunyatseni*, **42(5)**, 105-108 (2003) (in Chinese).

4. West, T. O., & Marland, G. A synthesis of carbon sequestration, carbon emissions, and net carbon flux in agriculture: comparing tillage practices in the United States. *Agric. Ecosyst. Environ.* **91(1),** 217-232 (2002).

5. Li, B., Zhang, J.B., & Li, H.P. Research on spatial-temporal characteristics and affecting factors decomposition of agricultural carbon emission in China. *China Popul. Resour. Environ.* **21 (8)**, 80-86 (2011) (in Chinese).

6. Oak Ridge National Laboratory https://www.ornl.gov (2016).

7. Institute of Resource, Ecosystem and Environment of Agriculture in Nanjing Agricultural University (IREEA) http://ireea.njau.edu.cn (2016).

8. Zhao, R. Q., & Qin, M. Z. Temporospatial variation of partial carbon source/sink of farm land ecosystem in coastal China. *J. Ecol. Rural Environ.* **23(2)**, 1-6 (2007) (in Chinese).

9. Duan, H. P., Zhang, Y., Zhao, J. B., & Bian, X. M. Carbon footprint analysis of farmland ecosystem in China. *J. Soil Water Conserv.* 2**5(5)**, 203-208 (2011) (in Chinese).

10. Wu, X. R., Zhang, J. B., Tian, Y., & Li, P. Provincial agricultural carbon emissions in China: Calculation, performance change and influencing factors. *Resour. Sci.* **36**, 129-138 (2014) (in Chinese).

11. Intergovernmental Panel on Climate Change (IPCC). IPCC Guidelines for National Greenhouse Gas Inventories https://www.ipcc-ggip.iges (2006).

12. Abulizi, A. et al. Land-use change and its effects in Charchan Oasis, Xinjiang, China. *Land Degrad. Dev*. **28(1),** 106-115 (2017).

13. Eziz, M., Yimit, H., Tursun, Z., & Rusuli, Y. Variations in ecosystem service value in response to Oasis land-use change in Keriya Oasis, Tarim Basin, China. *Nat. Areas J.* **34(3),** 353-364 (2014).
